# Evaluation of the Cortical Silent Period of the Laryngeal Motor Cortex in Healthy Individuals

**DOI:** 10.3389/fnins.2017.00088

**Published:** 2017-03-07

**Authors:** Mo Chen, Rebekah L. S. Summers, George S. Goding, Sharyl Samargia, Christy L. Ludlow, Cecília N. Prudente, Teresa J. Kimberley

**Affiliations:** ^1^Divisions of Physical Therapy and Rehabilitation Science, Department of Rehabilitation Medicine, School of Medicine, University of MinnesotaMinneapolis, MN, USA; ^2^Department of Otolaryngology-Head and Neck Surgery, University of MinnesotaMinneapolis, MN, USA; ^3^Department of Communication Sciences and Disorders, University of Wisconsin River Falls CampusRiver Falls, WI, USA; ^4^Department of Communication Sciences and Disorders, James Madison UniversityHarrisonburg, VA, USA

**Keywords:** Transcranial magnetic stimulation, TMS, larynx, motor cortex excitability, fine wire electrode, cortical silent period, cSP

## Abstract

**Objective:** This work aimed to evaluate the cortical silent period (cSP) of the laryngeal motor cortex (LMC) using the bilateral thyroarytenoid (TA) muscles with transcranial magnetic stimulation (TMS).

**Methods:** In 11 healthy participants, fine-wire electromyography (EMG) was used to record bilateral TA muscle responses to single pulse TMS delivered to the LMC in both hemispheres. Peripheral responses to stimulation over the mastoid, where the vagus nerve exits the skull, were collected to verify the central origin of the cortical stimulation responses by comparing the latencies.

**Results:** The cSP duration ranged from 41.7 to 66.4 ms. The peripherally evoked motor-evoked potential (MEP) peak occurred 5–9 ms earlier than the cortical responses (for both sides of TAs: *p* < 0.0001) with no silent period. The right TA MEP latencies were earlier than the left TA responses for both peripheral and cortical measures (*p* ≤ 0.0001).

**Conclusion:** These findings demonstrate the feasibility of measuring cSP of LMC based on intrinsic laryngeal muscles responses during vocalization in healthy volunteers.

**Significance:** The technique could be used to study the pathophysiology of neurological disorders that affect TA muscles, such as spasmodic dysphonia. Further, the methodology has application to other muscles of the head and neck not accessible using surface electrodes.

## Introduction

The laryngeal motor cortex (LMC) plays a significant role in human voice and speech production (Henriquez et al., 2007; Simonyan et al., 2009; Ludlow, 2015). However, its functional organization and interactions with other brain regions in both healthy humans and patients with neurological voice and speech disorders warrants further investigation (Simonyan and Horwitz, 2011). Specifically, for example, it is unknown how neurotransmitters, such as dopamine and gamma-aminobutyric acid (GABA) influence and modulate the human LMC network during voice and speech production and how these neurotransmitters are altered in people with neurological voice and speech disorders. This information is crucial in identifying the target brain regions for the development of new neuropharmacological options to modulate the LMC activity in patients with neurological voice problems, such as spasmodic dysphonia (Ludlow et al., 2008).

Transcranial magnetic stimulation (TMS) provides an important non-invasive way to evaluate the corticospinal excitability in a wide range of healthy and disease populations (for review: Eldaief et al., 2013). If the TMS stimulus is delivered to the motor cortex, a response in the peripheral muscles can be measured using electromyography (EMG). The response is defined as a motor-evoked potential (MEP). By assessing the MEPs induced by single or paired pulse techniques, TMS can be used to evaluate different aspects of cortical excitability, e.g., motor threshold, MEP latency and amplitude, and the cortical silent period (cSP) (Hallett, 2007).

Among these excitability measures, cSP is a widely adopted and highly reliable way to evaluate motor cortex excitability and its responses to neuromodulation (Wolters et al., 2008; Chen et al., 2015). Since the first reported cSP evoked by TMS (Calancie et al., 1987), cSP has been studied extensively in physiological and pathological conditions. Currently, it is thought that the cSP reflects intracortical inhibitory process mediated by GABA_A_ (Paulus et al., 2008) and GABA_B_ receptors (Wolters et al., 2008). This unique feature makes the cSP a powerful tool to non-invasively probe the GABA receptor mediated intracortical inhibitory process, especially in people with pathological conditions, such as dystonia (Siebner et al., 1998). Significantly shorter cSP has been reported in hand muscles in people with focal hand dystonia (Tinazzi et al., 2005; Kimberley et al., 2009), facial muscles in people with cranial dystonia (Currà et al., 2000) and hand muscles in people with spasmodic dysphonia (Samargia et al., 2014). Thus, cSP has the potential to reveal the abnormal inhibition in neurological disorders and may serve as a biomarker to help with diagnosis and early intervention.

Testing of the cSP can be performed by applying a single suprathreshold TMS pulse to the motor cortical representation of a tonically preactivated target muscle, producing a period of EMG silence in contralateral target muscles (Wolters et al., 2008). The hand region of the motor cortex has been the primary location of cSP testing due to the ease of accessing the corresponding muscles, such as first dorsal interosseous (FDI) or abductor pollicis brevis. The EMG signal from these peripheral muscles is large in amplitude with sufficient latency from the TMS pulse to make it easily identifiable and unaffected by TMS artifact. Furthermore, these muscles are easily accessible with surface electrodes which summate MEPs from a large number of motor units, making the EMG signal less sensitive to noise or the firing of individual motor unit. However, in order to evaluate the cSP from the deep muscles (i.e., the intrinsic laryngeal muscles in people with spasmodic dysphonia), intramuscular electrodes, i.e., fine-wire or needle electrodes, must be used. Signals from intramuscular electrodes are more difficult to assess than surface electrodes (Konrad, 2005). Reasons for the difficulties are as follows: (1) Electrode placement is technically difficult. (2) The position of the two fine-wire electrodes cannot be altered once inserted. If the initial insertion is not accurate another insertion is required; and (3) Fine-wire or needle electrodes summate evoked potentials from fewer motor units than surface electrodes, reducing the MEP amplitude and making it difficult to differentiate the MEP from spontaneous firing of intrinsic motor units. A challenge specific to measurements from the LMC is that the EMG electrode location is close to the stimulation site, which results in a large stimulus artifact. When the stimulation artifact is lengthened due to amplifier saturation, it is difficult to identify MEPs with early latencies. Finally, the LMC brain representation is relatively small compared to regions, such as the hand and, therefore, stimulation location may be challenging to locate.

Overcoming these difficulties, several groups have studied the MEP responses to TMS over the LMC. MEP latency and amplitude values from the cricopharyngeal sphincter muscles during cortical stimulation have been reported (Ertekin et al., 2001); single pulse responses from the cricothyroid muscles were also reported (Espadaler et al., 2012; Rogić Vidaković et al., 2015). Another laryngeal intrinsic muscle, the thyroarytenoid (TA), which directly controls the vocal folds by modulating vocal-fold tension when opposed by other intrinsic muscles, is highly relevant for the pathophysiology of speech related neurological disorders, i.e., spasmodic dysphonia and voice tremor (Ludlow, 2005; Simonyan et al., 2009; Simonyan and Horwitz, 2011). However, the TA muscle has only been used to evaluate the latency of MEPs during cortical stimulation to LMC (Khedr and Aref, 2002; Rödel et al., 2004). These latencies were relatively early (≤10 ms) and some were close to TA latencies found with peripheral stimulation over the mastoid (Sims et al., 1996). Moreover, the excitability of the LMC as measured by cSP in TA muscles has not been investigated. Considering that investigation of the excitability of corticobulbar projections to the TA would be relevant for understanding the neural controls of voice production in both healthy and pathological conditions, the purpose of the current study was to assess the excitability of the LMC by measuring the cSP in the TA muscles. The use of TMS with fine-wire electrodes to measure the cSP of the LMC will provide a tool to evaluate the GABA receptor mediated inhibition process. This will further lead to a better understanding of the pathophysiology of disorders of the larynx i.e., spasmodic dysphonia and voice tremor.

## Methods

### Participants

Data from eleven healthy participants (mean age, 54 ± 7.4 years; 3 females) were collected. Participants gave written, informed consent prior to participation according to the Declaration of Helsinki (World Medical Association, 2013). The study was approved by the Clinical and Translational Science Institute and the Institutional Review Board of the University of Minnesota.

### Devices

Transcranial magnetic stimulation (TMS) pulses were delivered using a 70 mm figure-of-eight coil connected to the Bistim^2^ and 200^2^ stimulator set (The Magstim Company Ltd, UK). Two pairs of fine-wire electrodes were connected to two bi-polar active pre-amps (Y03-002, Motion Lab Systems, Inc., Baton Rouge, LA) powered by two 9-volt batteries. EMG signals were amplified with a gain of x300, passed through a band-pass filter (15–2000 Hz), and digitized by a 24-bit analog-to-digital converter (NI9234, National Instruments Co., Austin, TX) in AC coupling mode (0.5 Hz) with the sampling rate of 6.4 k Hz. All data were collected and stored using a custom data acquisition program written with LabVIEW (V2012, National Instruments, Austin, TX) on a laptop computer (Latitude, Dell Co., Ltd, Round Rock, TX) which was also used to monitor real-time EMG activity.

### Experiment procedures

Participants were seated comfortably in a reclined armchair with the subject tracker band of a frameless stereotactic neuronavigation system (BrainSight, Rogue Research Inc., Canada) attached to their forehead. The hand region was assessed prior to the laryngeal area.

#### Hand region assessment

Hand region excitability was evaluated by using surface electrodes (6030-TP, Nicolet, CA, USA) attached to right hand FDI muscle. The experiment procedure was the same as previously published protocols (Chen et al., 2015; Rossini et al., 2015). Briefly, the resting motor threshold was determined as the lowest intensity that generated MEPs with the peak-to-peak amplitude >50 μV in 5 out of 10 consecutive trials. The hotspot was the location of the coil where the resting motor threshold was determined. The 1-mV threshold was determined by using a similar protocol with the MEP amplitude response ≥1 mV. The 1-mV threshold intensity was used as the initial stimulation intensity for the LMC assessment.

#### Laryngeal region assessment

##### Skin preparation

Skin around the area of the laryngeal prominence was cleaned using alcohol wipes. A topical anesthetic cream (LMX 4% Lidocaine, Ferndale Laboratories, Inc., Ferndale, MI) was applied to the cleaned area. After approximately 15 min, a numbing agent (Xylocaine, 2% lidocaine HCL and epinephrine, 1:100,000, Professional Veterinary Laboratories, NB, Canada) was injected into the skin of the numbed region.

##### Electrodes placement

**Figure 1:**
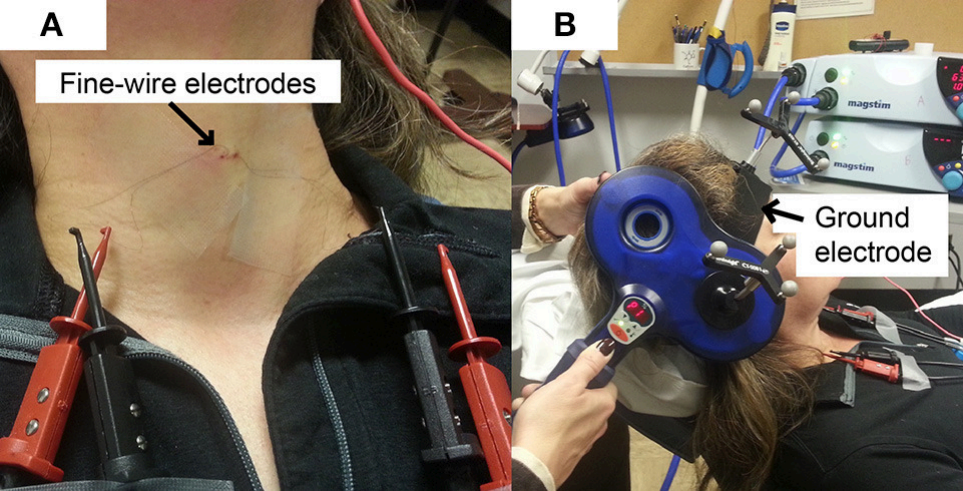
**Experimental Set up. (A)** Fine-wire electrodes. There were two channels (pairs of fine-wires) inserted into bilateral thyroarytenoid muscles; **(B)** Ground electrode. The strap ground electrode was placed under the BrainSight subject tracker band.

A 30 mm, 27 gauge needle loaded with a pair of fine-wire hooked electrodes (#019-772800, Nicolet Co., Middleton, WI) was inserted into left and right TA muscle by an experienced otolaryngologist following standard procedures for laryngeal EMG (Hirano and Ohala, 1969). Using a percutaneous approach, the needle was passed through the cricothyroid membrane at an angle off midline but medial to the ipsilateral inferior tubercle, to directly enter the TA muscle while avoiding the airway. During insertion, the electrodes were connected to an audio monitor (Grass AM10, Natus Medical Incorporated Co., San Carlos, CA) to allow monitoring of muscle activity in real-time. After the location was confirmed, the needle was removed leaving the fine-wires in the TA muscles (Figure 1A). A silver-silver chloride strap with a Velcro fastener (TD-431, Discount Disposables, St. Albans, VT) was attached to the participant's forehead serving as a ground (Figure 1B).

##### Peripheral stimulation

**Figure 2:**
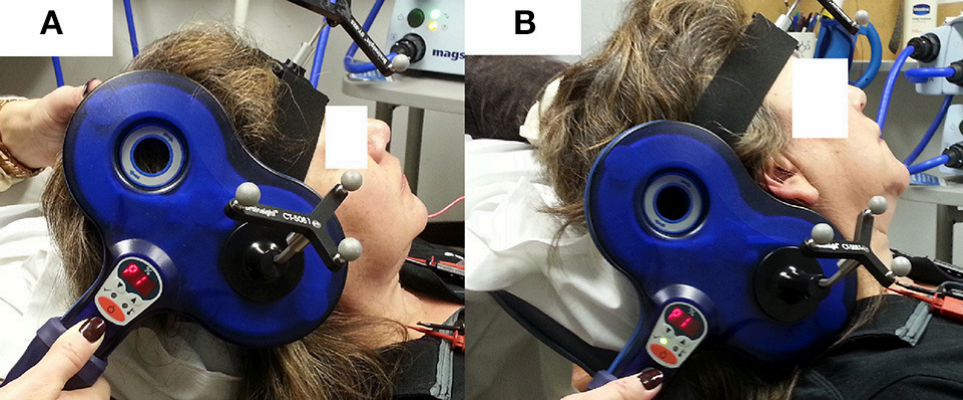
**TMS coil placement for laryngeal motor cortex (LMC) and peripheral stimulation. (A)** Coil position and angle for LMC stimulation (cSP test); **(B)** coil position and angle for peripheral stimulation.

Peripheral stimulation was delivered over the mastoid to: (1) confirm electrode placement in the TA muscles, (2) determine peripheral stimulation response latency, and (3) contrast with the cortical stimulation responses to ensure that there was not stimulus spread to the vagus nerve during cortical stimulation. Ten peripheral stimulation trials were collected; 5 at rest and 5 during a production of sustained /i/. The TMS coil was placed over the mastoid bone to activate the vagus nerve. Placement was tangential to the tip of mastoid bone in a posterior-anterior direction (Figure 2B). The center of the coil was located above the mastoid (the exit of vagus nerve from the skull through the jugular foramen) behind the ear. This placement is consistent with previous studies (Sims et al., 1996; Khedr and Aref, 2002). The stimulation intensity was set to 40% of maximum TMS output. Preliminary work determined that 40% was the lowest intensity that consistently generated peripheral evoked potentials in all participants with very little variation. Higher stimulation intensities induced facial muscle contractions during the experiment and caused the magnetic field spread to the electrode area, resulting in amplifier saturation.

##### Cortical stimulation

An anatomical T1 magnetic resonance image (MRI) with high-resolution (1 × 1 × 1 mm^3^) was acquired on a separate day prior to the TMS experiment visit. The image was imported into the neuronavigation system to guide the localization of the LMC in the primary motor cortex (M1). The location of the LMC, as determined by Simonyan et al. (2009), was used to help direct neuronavigation of initial coil placement on each participant's skull. This location is reported as approximately 0.5–1 cm anterior and 2–4 cm lateral to the hotspot determined in the afore-determined hand region assessment (Simonyan et al., 2009). This LMC location is similar to the 2 cm anterior and 4–8 cm lateral to the Cz EEG electrode position in the 10–20 system reported by Ertekin et al. (2001). The coil was held tangential to the skull over the targeted area in a posterior-anterior direction parallel to the midline (Figure 2A) and was systematically moved in an approximate 1 cm grid. The procedure was monitored by the neuronavigation system in real time. Initially, the intensity used for single TMS pulses was the 1-mV threshold for the FDI muscle as determined in the aforementioned hand region assessment; however, in most cases, the intensity was increased to induce an MEP in TA muscles. Conventional motor threshold determination protocol was attempted with the observation that the MEP amplitude was not modulated with TMS intensity within participant's acceptable/comfortable range (80% of the maximum stimulator output). However, a silent period was clearly observed following the superimposed MEP when a given TMS intensity was reached. Thus, cSP threshold was defined as the lowest TMS intensity that elicits a cSP in 5 out of 10 consecutive trials. The location that corresponded to the lowest stimulation intensity (cSP threshold) was used as the LMC “hotspot” in the following experiment procedures. The hotspot coordinates were recorded by the neuronavigation system in MNI standard space using template ICBM152 with 1 mm^3^ resolution (Mazziotta et al., 2001).

##### Outcome measure

cSP was the outcome measure for LMC excitability. The cSP threshold was used as the stimulation intensity. Single pulse cortical stimulations were performed during vocalization of sustained /i/. Participants were instructed to produce a comfortable pitch and volume of vocalization that was kept similar throughout the trials. The single pulse was applied approximately 1 s after initiation of the sustained vocalization. Participants were instructed to relax approximately 2 s after the pulse was delivered. In all participants, cSP threshold and cSP responses were first collected in the left hemisphere followed by the right hemisphere. Fifty trials of bilateral cSP responses in left and right TA muscles were collected in response to stimulation in both hemispheres. Given the low signal-to-noise ratio (SNR) with the fine wire responses, 50 trials were collected and traces were averaged to cancel out noise and increase the SNR. Average MEP amplitude was also calculated.

### Data processing

The cSP data were first averaged and rectified. Then, a 10-ms moving standard deviation (SD) window was applied to generate an SD curve of the signal. The average value of the SD curve during baseline (100–5 ms before stimulus) was calculated as the baseline contraction level. This value was used to define the offset of the cSP when the signal returned to pre-stimulus level. The onset of the cSP was defined as the time point of the stimulus. cSP duration was calculated by subtracting the onset from the offset of the cSP (Wolters et al., 2008; Chen et al., 2015). The MEP peak latency for both cortical and peripheral stimulation was defined as the duration between the stimulation artifact and the first peak of the MEP. The latency of the MEP to peripheral stimulation was calculated from each trial of stimulation; the latency of the MEP from cortical stimulation was identified from the average of 50 trials. The advantage of using the average of 50 trials was that it overcame any obscured response secondary to the active contraction of the TA muscle. MEP amplitude was calculated by the following procedures: in the averaged cSP trace, MEP was first identified within the range of 10–30 ms after the stimulus artifact. Then the peak-to-peak amplitude was extracted to represent the corresponding participant's MEP amplitude.

### Data analysis

Motor-evoked potential (MEP) latencies for peripheral stimulation under both active and resting conditions, and cortical stimulation for each hemisphere were compared. All data were normally distributed as determined by the Shapiro-Wilk W test. Multi-factor ANOVAs were used to examine the following three hypotheses to confirm the cortical nature of the evoked responses. (1) Cortical latencies are longer than peripheral latencies for the TA muscle on the same side. (2) MEP latencies from the left TA are longer than the right TA within the same type of stimulation (due to the longer length of the left recurrent laryngeal nerve Atkins, 1973). (3) There are no differences in latency for TA muscle on the same side when tested under different conditions. This hypothesis was tested with two sub-hypotheses: (3a) There are no differences in TA latency between resting and active with peripheral stimulation; (3b) there are no differences in TA latency between the cortical stimulation on the left and right hemispheres. These three hypotheses have to be tested separately because the cortical and peripheral data are not balanced. That is, all cortical data were collected during active contraction and peripheral data were collected under active and resting. Therefore, a three-step approach was used: for hypotheses 1 and 2 (cortical comparison), cortical and active peripheral data were tested by a two-way ANOVA with “stimulation type” (cortical vs. peripheral) and “TA side” (left vs. right) as interaction factors. For hypotheses 2 (peripheral comparison) and 3a, peripheral data were tested by a two-way ANOVA with “TA state” (rest vs. active) and “TA side” (left vs. right) as interaction factors. For hypothesis 3b, cortical data were tested by a two-way ANOVA with “stimulation side” (left vs. right) and “TA side” (left vs. right) as interaction factors. The significance level was set as *p* < 0.05 for all tests.

## Results

No serious or unexpected adverse effects were reported. All participants tolerated the procedures with expected adverse events including skin soreness (*n* = 4), bruising (*n* = 1), and a tender throat (*n* = 2). Cortical stimulation intensities (percentage of the maximum stimulator output) are listed in Table 1. Average coordinates (MNI standard space) of the LMC hotspot were *x* = −56, *y* = −3, *z* = 36 in the left hemisphere and *x* = 56, *y* = 3, *z* = 37 in the right hemisphere. Individual coordinates are listed in Supplementary Table 1.

**Table 1:** **Participant information**.

**Participant**	**Sex**	**Age (years)**	**Stimulation intensity (% MSO)**	
			**L-cortical**	**R-cortical**
1	F	42	60	60
2	F	56	63	63
3	F	49	63	63
4	M	62	57	61
5	M	58	64	65
6	M	67	52	52
7	M	60	67	58
8	M	56	55	52
9	M	47	60	60
10	M	49	60	60
11	M	46	50	52

*MSO, maximum stimulator output; L, left hemisphere cortical stimulation; R, right hemisphere cortical stimulation*.

**Figure 3:**
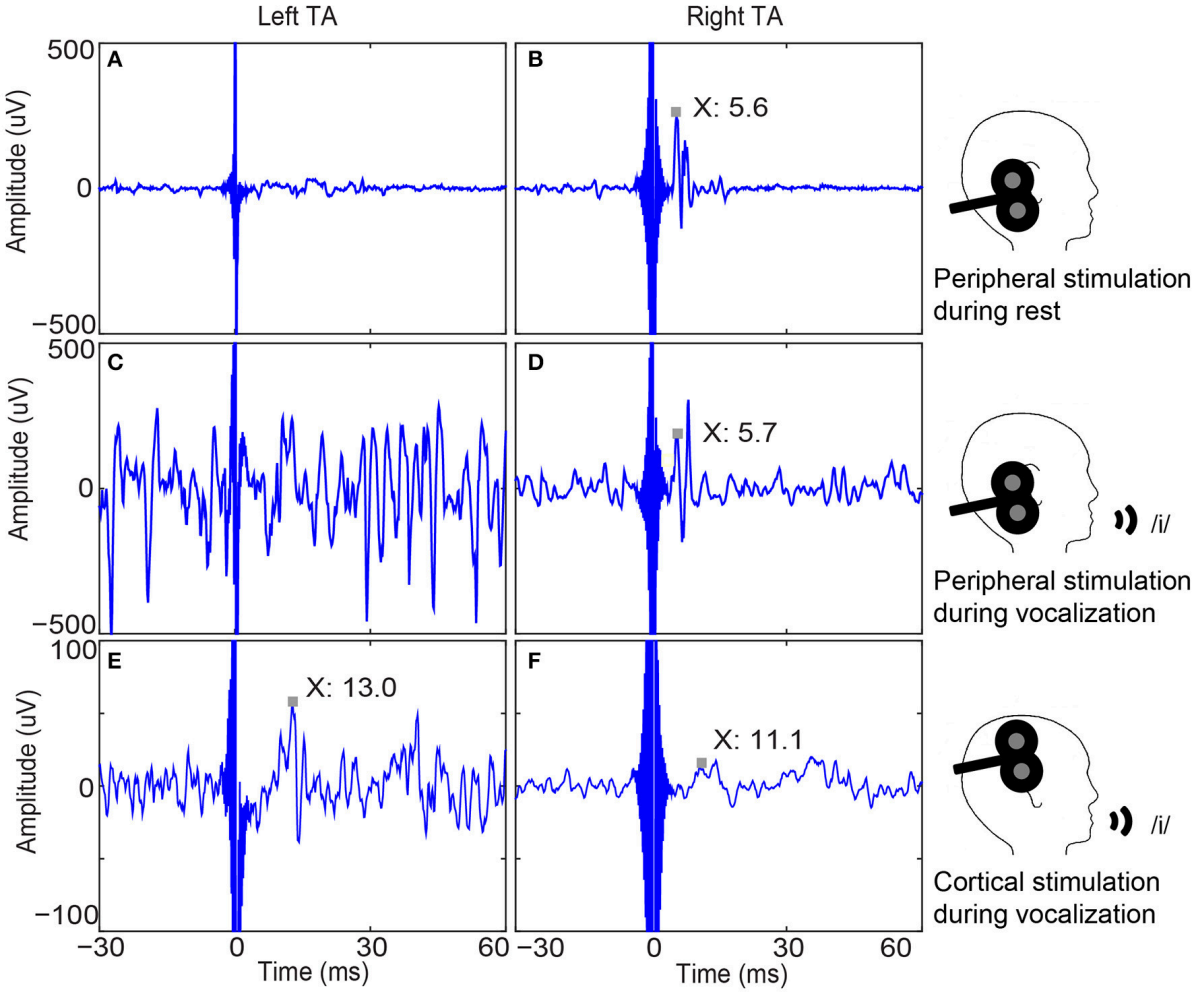
**Bilateral thyroarytenoid (TA) muscle responses to right peripheral and cortical stimulation in one participant. (A)** Left TA recording without a response to right peripheral stimulation during rest (sample trace); **(B)** Right TA response to right peripheral stimulation during rest (MEP latency is 5.6 ms.); **(C)** Left TA recording without a response to right peripheral stimulation during vocalization (sample trace); **(D)** Right TA response to right peripheral stimulation (MEP peak latency is 5.7 ms.); **(E)** Average of 50 left TA responses to right cortical stimulation (MEP peak latency is 13.0 ms); **(F)** Average of 50 right TA responses to right cortical stimulation (MEP peak latency is 11.1 ms). Note the unilateral (ipsilateral) responses to peripheral stimulation and bilateral responses to cortical stimulation, which demonstrates the validity of cortical responses. MEP: motor evoked potential.

**Figure 4:**
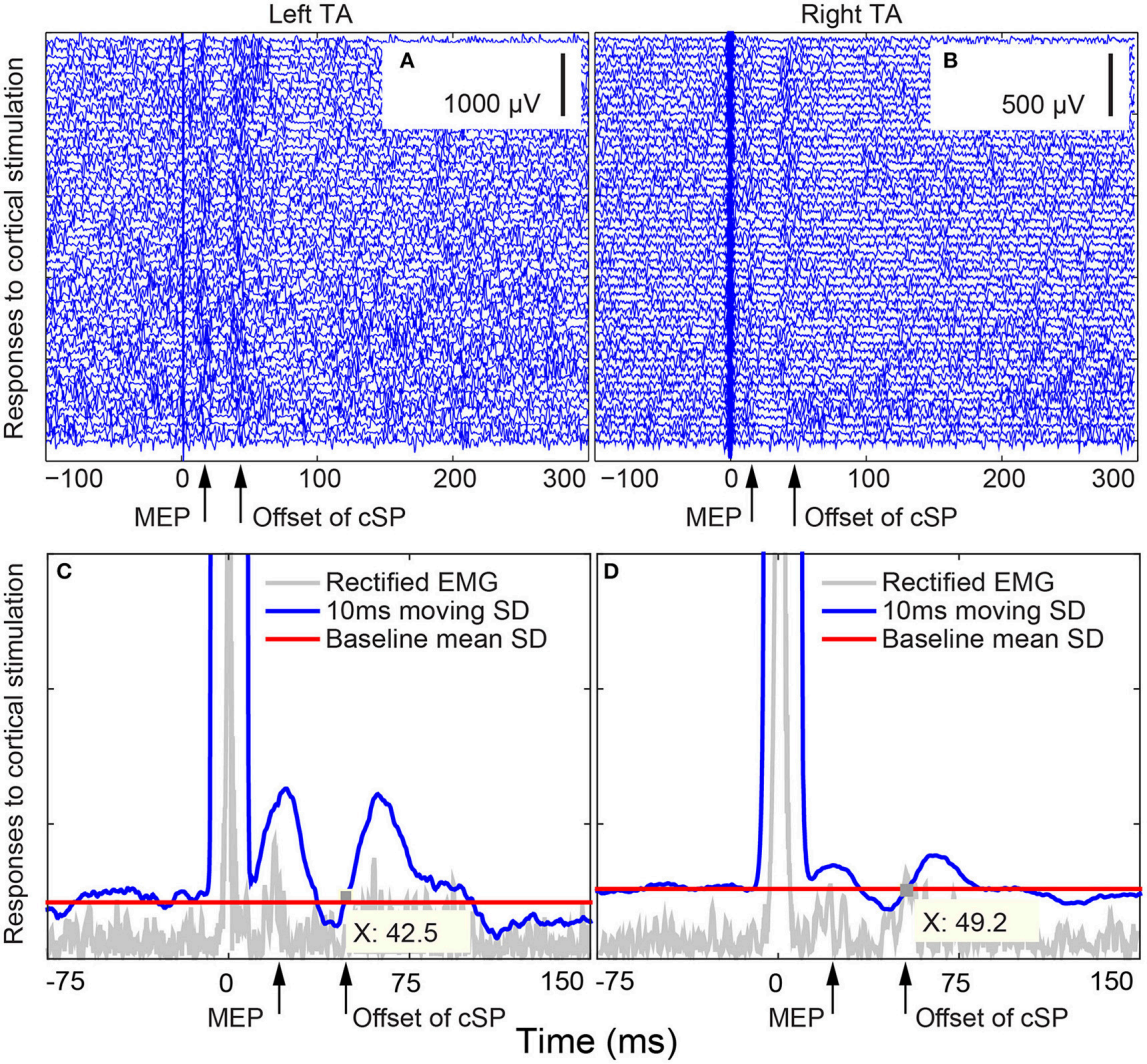
**Bilateral thyroarytenoid (TA) motor evoked potential (MEP) responses to right cortical stimulation during voice production in Subject 02. (A)** 50 individual traces of left TA responses to right cortical stimulation; **(B)** 50 individual traces of right TA responses to right cortical stimulation; **(C)** cSP from the left TA during right cortical stimulation (offset X at 42.5 ms); **(D)** cSP from the right TA during right cortical stimulation (offset X at 49.2 ms). The cSP moving average was calculated based on the average of the 50 trials. The stimuli were delivered at 0 ms. cSP: cortical silent period.

Peripheral stimulation induced unilateral (ipsilateral) responses of shorter latencies both during rest (left TA: 9.1 ± 2.2 ms; right TA: 6.9 ± 1.4 ms) and active contraction (left TA: 9.3 ± 2.2 ms; right TA: 6.8 ± 1.3 ms). Individual responses are listed in Supplementary Table 2. Importantly, no silent period was observed after active peripheral stimulation. Cortical stimulation evoked bilateral responses with silent periods. Average MEP latencies for left hemisphere cortical stimulation were 15.6 ± 2.3 ms in the left TA and 13.1 ± 2.0 ms in the right TA; average MEP latencies for right hemisphere cortical stimulation were 15.5 ± 2.8 ms in the left TA and 13.1 ± 2.3 ms in the right TA. The cSP duration and MEP peak latency values are listed in Table 2. Average cSP duration from left hemisphere cortical stimulation was 53.7 ± 7.8 ms in the left TA and 52.8 ± 7.3 ms in the right TA; average cSP duration from right hemisphere cortical stimulation was 53.4 ± 7.8 ms in the left TA and 54.5 ± 5.9 ms in the right TA. The average MEP amplitude from the left hemisphere cortical stimulation was 89.2 ± 80.0 μV in the left TA and 142.1 ± 142.5 μV in the right TA; the average MEP amplitude from the right hemisphere cortical stimulation was 110.6 ± 106.4 μV in the left TA and 196.3 ± 194.0 μV in the right TA. Individual MEP amplitude details are listed in Table 2. Samples of MEP responses are shown in Figure 3 and cSP responses during right cortical stimulation are shown in Figure 4.

**Table 2:** **Cortical stimulation MEP latency, MEP amplitude and CSP duration**.

**Participant**	**MEP latency (ms)**				**MEP amplitude (μV)**				**CSP duration (ms)**			
	**Left hemisphere**		**Right hemisphere**		**Left hemisphere**		**Right hemisphere**		**Left hemisphere**		**Right hemisphere**	
	**L-TA**	**R-TA**	**L-TA**	**R-TA**	**L-TA**	**R-TA**	**L-TA**	**R-TA**	**L-TA**	**R-TA**	**L-TA**	**R-TA**
1	12.3	8.9	11.9	8.6	32.7	296.2	33.5	34.1	51.4	44.8	50.3	46.4
2	12.5	11.4	13.0	11.1	81.0	26.5	95.5	34.3	43.3	44.2	42.5	49.2
3	16.1	13.4	14.2	12.7	26.3	105.1	40.9	100.0	53.6	53.4	53.0	53.0
4	16.3	13.9	15.9	11.6	58.1	23.3	12.6	92.1	50.2	47.7	53.8	55.3
5	16.6	13.6	16.3	12.7	10.0	10.3	7.4	3.9	65.3	62.8	64.7	60.9
6	17.8	13.8	15.2	13.8	26.1	10.7	71.2	16.7	48.4	46.1	48.4	49.2
7	13.9	13.0	13.8	11.7	59.1	398.6	128.8	467.3	41.7	46.7	43.9	46.9
8	N/A	15.5	N/A	15.5	N/A	383.1	N/A	322.6	N/A	55.9	N/A	56.4
9	13.4	10.3	15.3	13.9	246.8	101.0	270.3	314.5	60.0	59.2	63.6	63.1
10	19.7	15.5	22.5	17.0	128.0	165.7	344.0	606.2	63.6	66.4	64.4	63.8
11	17.3	14.5	17.0	15.5	224.5	42.4	101.8	167.3	59.5	53.6	53.4	55.6

*Values are calculated from averaged trace. MEP amplitude, peak to peak amplitude; MEP, motor evoked potential; cSP, cortical silent period; TA, thyroarytenoid; L-TA, left TA; R-TA, right TA; N/A, no signal was observed from the corresponding channel*.

The two-way ANOVA tested cortical and active peripheral data with “stimulation type” and “TA side” as interaction factors with significant effects in “stimulation type” [*F*_(1, 59)_ = 113.4894, *p* < 0.0001] and “TA side” [*F*_(1, 59)_ = 18.0334, *p* < 0.0001] factors. No significant effect was found in the interaction of the two factors [*F*_(1, 59)_ = 0.0140, *p* = 0.9062]. This indicates that the cortical MEP latencies were longer than the peripheral MEP latencies regardless of TA side; and the left TA MEP latencies were longer than the right TA MEP latencies regardless of stimulation type. These results support hypotheses 1 and 2 (cortical comparison).

The two-way ANOVA test on peripheral data showed a significant effect with “TA side” [*F*_(1, 38)_ = 18.2670, *p* = 0.0001] as a significant factor. No significant effects were found in either “TA state” [*F*_(1, 38)_ = 0.0011, *p* = 0.9734] or the interaction of the two factors [*F*_(1, 38)_ = 0.0213, *p* = 0.8849]. This indicates that the left TA peripheral MEP latencies were longer than the right TA peripheral MEP latencies regardless of the “TA state” (rest vs. active) and that there was no difference in peripheral MEP latencies between the two TA states (rest and active). These findings support hypotheses 2 (peripheral comparison) and 3a.

**Figure 5:**
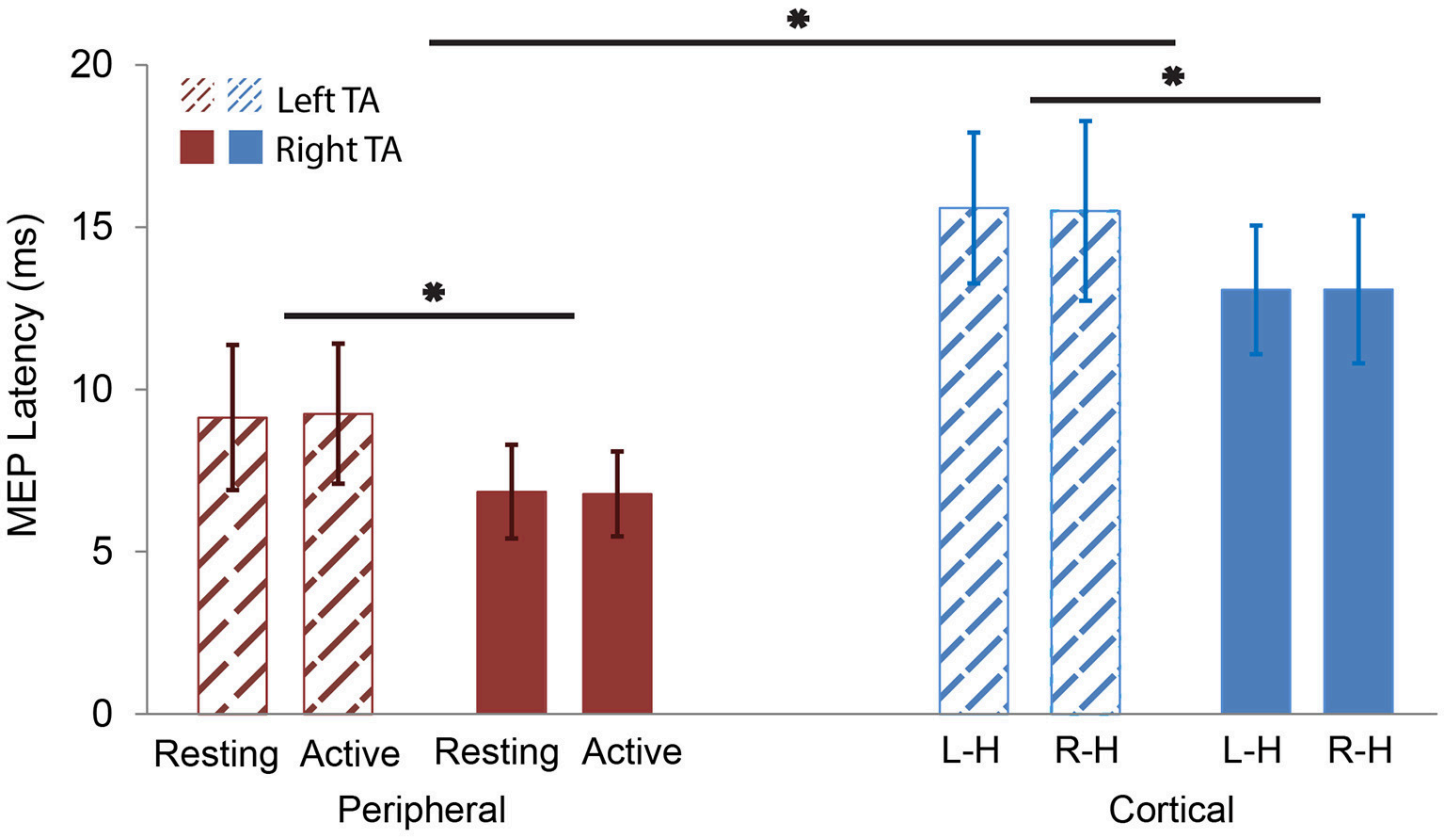
**MEP latency comparisons. Red color represents peripheral stimulation responses (left)**. Blue color represents cortical stimulation responses **(right)**. Thatched represents left TA responses. Solid represents right TA responses. The MEP latencies of left TA were significantly longer than the right TA in both peripheral and cortical stimulation. The MEP latencies of the cortical stimulation were significantly longer than the peripheral one in both left and right TAs. L-H: cortical Left Hemisphere; R-H: cortical Right Hemisphere. ^*^p < 0.05.

The final two-way ANOVA test on cortical data with “stimulation side” and “TA side” as interaction factors showed significant effects in “TA side” [*F*_(1, 38)_ = 10.5621, *p* = 0.0024]. No significant effects were observed in either “stimulation side” [*F*_(1, 38)_ = 0.0027, *p* = 0.9592] or the interaction between the two factors [*F*_(1, 38)_ = 0.0048, *p* = 0.9450]. This indicates that in cortical stimulation, left TA latencies were longer than right TA latencies regardless of stimulation side (hemispheres) and that there was no difference between cortical MEP latencies evoked by stimulation to different hemispheres. These results support hypothesis 3b (Figure 5).

## Discussion

In this work we evaluated the feasibility and validity of testing cortical excitability of LMC with the cSP measurement using fine-wire electrodes inserted into TA muscles. TMS evoked MEP responses were confirmed as cortical in origin by observation of bilateral responses secondary to cortical stimulation, the occurrence of a silent period, and contrasting the MEP peak latencies of peripheral and cortical responses. These results suggest that the cSP may be used as a measure of cortical excitability for the LMC. To our knowledge, these cSP findings were the first report of cSP as a measure of the cortical excitability of the LMC.

### Cortical vs. peripheral responses

The significant difference in MEP latencies between the peripheral and cortical stimulation confirmed that the cSP and MEP were generated cortically. No early response in the cortical data was observed at a similar latency as the peripheral stimulation, confirming that there was no current spread to the peripheral vagus nerve during cortical stimulation.

### Cortical silent period

The cSP duration for the TA muscles reported in this work were much shorter than previously reported values collected from the hand muscles (Chen et al., 2015). Previous studies have reported that the cSP duration is longest in small hand muscles (up to 300 ms), shorter in leg muscles (up to 100 ms), proximal arm muscles (Roick et al., 1993), axial muscles (Ferbert et al., 1992; Lefaucheur and Lofaso, 2002; Lefaucheur, 2005), facial muscles (the triangularis, range 69–169 ms, and orbicularis oculi, range 68–111 ms) (Werhahn et al., 1995; Paradiso et al., 2005) and the tongue (64.2 ± 4.5 ms at 50% maximum stimulator output) (Katayama et al., 2001). Our reported values are in line with other cranial muscles. Of note, as the brain volume decreases with age, the cSP duration gets shorter (Silbert et al., 2006). Considering the age range (54 ± 7.4 years) of participants in this study, this could also be part of the reason for the early cSP offset observed.

The cSP duration values collected from the left and right TA regardless of hemisphere stimulation were similar, the differences were within 2 ms. This symmetry is consistent with previously reported results of the low interhemispheric difference of cSP duration (usually <10 ms) in healthy subjects (Wolters et al., 2008). Although the motor cortical representation of the cSP is lateralized to the contralateral motor cortex for distal limb muscles, for axial muscles the cSP shows a more bilateral distribution (Wolters et al., 2008) which is consistent with our findings here.

Of note, MEP amplitude was not observed to modulate with changes in TMS intensity. This may be due to the small summation area of fine-wire electrodes, in contrast to surface electrodes on larger muscles, which do demonstrate changes in MEP size with different TMS stimulation intensities. This lack of modulation likely has little effect on cSP values (Rossini et al., 1994), but may render paired pulse responses or stimulus response curve measures unreliable. Also, given that the TA muscles are always active during breathing (Kuna et al., 1988), this makes resting measures difficult to reliably execute. Furthermore, it has been reported that the cSP induced by TMS can be elicited at lower stimulation intensities in the absence of a preceding MEP (Davey et al., 1994; Classen and Benecke, 1995). This low stimulation intensity advantage helps with the suppression of the stimulus artifact due to saturation of the amplifier, especially when the two inputs of the differential amplifier are not balanced, which is very likely with fine-wire electrodes, and the ground impedance is not low enough. Altogether, these inherent limitations of the laryngeal measurements suggest that cSP may be the ideal cortical excitability measure.

### Latency of motor evoked potential

The MEP latencies for peripheral and cortical stimulation measured in the current study were longer than those previously reported (Thumfart et al., 1992; Khedr and Aref, 2002). In those investigations, the cortical latency was reported as 9.6 ms in the left TA and 11.1 ms in the right TA contralateral to stimulation; and 9.3 and 9.5 ms in the TA ipsilateral to cortical stimulation. They also reported shorter peripheral latencies (between 2.7 and 6 ms), 1–7 ms shorter than the 3.2–13.2 ms range as we report here. These differences may be due to different definitions of the MEP latency. Khedr and Aref (2002) defined MEP latency as the interval between the stimulation artifact and the *onset* of the MEP. In this study, we measured the interval between the stimulation artifact and the first *peak* of the MEP. We chose this definition because the peak is more easily identified and less ambiguous than the MEP onset due to background EMG activity caused by breathing or voice activation. When compared with the studies using the same definition for MEP latency, our results are in agreement with others' reported values (Atkins, 1973; Rödel et al., 2004). Although it has been reported that the age can be a factor to cause a later onset of MEP (Eisen and Shtybel, 1990), in our findings no such a trend was observed.

Furthermore, the differences in latency between the left and right TA muscles (2–4 ms) are consistent with the previous reported values (Atkins, 1973; Thumfart et al., 1992; Sims et al., 1996; Khedr and Aref, 2002; Rödel et al., 2004), as are the latency differences between peripheral and cortical responses (4–6 ms) (Thumfart et al., 1992; Khedr and Aref, 2002). The consistency of our results with others supports the validity of this method.

### Methodological considerations

#### Stimulation artifact suppression

Key considerations regarding stimulation artifact suppression were applied for improved signal quality as follows: *First*, a large contact area of the ground electrode suppressed the stimulation artifact, which was essential when the stimulation intensity was high (>80% of the maximum output). We determined that this procedure significantly reduced amplifier saturation as illustrated in Figure 3. *Second*, the ground electrode should be located between the stimulation site and the acquisition site and as close to the acquisition site as possible. For the TA or other intrinsic larynx muscles, the forehead or chin were ideal areas to attach the ground electrode. Our testing revealed that participants preferred the head strap due to comfort. Additional ground electrodes can be attached to achieve even greater suppression of the stimulation artifact. *Also*, a relatively low gain (such as x300 or x100) along with a large dynamic range of the amplifier (e.g., using a power supply generated by 9-V batteries or greater) decreased the chances of amplifier saturation. However, the low gain was compensated for by a high quantification resolution analog-to-digital convertor, such as the 24-bit used here. This high quantification resolution helped to improve the signal-noise ratio and increased the quantification accuracy that could have been diminished by the low gain. *Last*, the battery power to the amplifier also had the benefit of reducing susceptibility to main noise (e.g., 50 or 60 Hz) without requiring a notch filter which should be avoided during EMG/MEP data acquisition because a notch filter would also filter out the informative components of the EMG/MEP within the 50–60 Hz range (Konrad, 2005).

#### Insertion of fine-wire electrodes

Fine-wire electrodes were chosen instead of needle electrodes because they adapt to the muscle movement. During our experiment, the participants were asked to vocalize “/i/” which contracts the muscles in the vocal folds. Rigid needle electrodes resist muscle contraction causing pain during vocalization and dry swallows. However, insertion accuracy of bi-polar fine-wire pair electrodes is critical to data quality. An experienced otolaryngologist inserted the electrodes in this experiment. In some participants, however, re-insertion was necessary after an insertion did not yield an EMG signal.

In some participants, the two TA muscles had different EMG signal amplitudes, as shown in Figure 3 (e.g., left > right). This occurred frequently likely due to differences in the insertion angle and depth of the fine-wire electrodes. With different placement in the muscles, the number and size of the motor units between the two poles of the fine-wire electrodes will vary, resulting in differences in the signal amplitude. For this reason, the traditional peak-to-peak motor evoked potentials may not be a valid measure for assessing cortical excitability. The use of cSP, however, diminishes the impact of this issue because the cSP is a temporal/duration measure, not an amplitude measure. Thus, the shape of the MEP has less effect on cSP duration than that of the MEP amplitude. This makes the cSP a useful method for quantifying cortical excitability when using fine-wire electrodes.

## Limitations

During data collection, we did not attempt to control TA muscle contraction level as a percent of maximum voluntary contraction (MVC), as is typically performed for hand muscles. The rationale for this decision was that to control the contraction level of TA muscles, both the pitch and the amplitude of vocalization need to be standardized. For the standardization of voice pitch, it is required that the full range of vocal pitch of each participant is measured (similar to the MVC measurement in the conventional TMS test) and, that all participants generate a normalized relative pitch (similar to the 20% of MVC in the conventional hand TMS test) during the experiment. The same procedures apply to the standardization of the amplitude. To control the two factors (pitch and amplitude) synchronously may be difficult for participants to follow, especially when there are fine-wires inserted into their larynx. Thus, in this work we used a compromised but practical method to standardize the TA muscle contraction: participants were instructed to vocalize with their comfortable and natural pitch and volume consistently in each trial. This methodology will allow for future comparisons with populations that have difficulty in controlling their voice, such as people with spasmodic dysphonia. Although the cSP values were likely unaffected by this limitation, as contraction strength of the target muscle does not significantly influence cSP duration (Haug et al., 1992; Inghilleri et al., 1993; Roick et al., 1993; Taylor et al., 1997; Wu et al., 2002; Säisänen et al., 2008). Of note, these previously reported studies were done in large peripheral muscles, i.e., FDI. It is possible that this characteristic is different when measured in intrinsic laryngeal muscles. Thus, it is worth further investigation and may be a useful addition in future experiments.

In the current study, the cSP threshold was used instead of the conventionally adopted 120–130% of resting motor threshold in previous cSP studies (Wolters et al., 2008). This was because that it was difficult to determine a resting motor threshold from the constantly-firing TA muscles. However, it has been reported that the MEP threshold and cSP threshold are usually related (Kimiskidis et al., 2005), suggesting that the stimulation intensity used (cSP threshold) was valid.

Cortical silent period (cSP) threshold and responses were collected first in the left hemisphere followed by the right hemisphere in all participants. Given that the fine-wire electrodes may change position due to swallowing or head rotation during the experiment, we collected data from the hemisphere with higher priority first. The left LMC has been reported to have a more dominant role than the right LMC in vocalization (Simonyan et al., 2009) thus, it was tested first.

Considering the invasive procedure of the fine-wire insertion during the experiment, there was no reliability re-tests attempted in the study design. Although according to previous studies, the intersession variability of cSP is typically <10% (Kukowski and Haug, 1991; Orth and Rothwell, 2004), but this is unknown for laryngeal data.

The MEP amplitudes reported were calculated based on the averaged trace of the 50 cSP trials. Conventionally, the MEP amplitude is calculated based on individual traces and then the average values are reported. However, given the noisy nature of the TA EMG and the relatively low amplitude of the MEP, it is difficult to determine MEP amplitude based on each individual trace. Using the averaged trace provides a clear background to distinguish the MEP from noise or random firing spikes.

## Conclusion

We assessed the excitability of LMC using the TMS evoked cSP in the TA muscles. The cSP duration was in line with other healthy cranial muscles, such as the tongue (Katayama et al., 2001). The responses were confirmed by contrasting the difference in the MEP peak latencies that were generated by cortical and peripheral stimulation, respectively. Given there are reports of the low intersession variability of the cSP duration in a given subject, typically <10% (Kukowski and Haug, 1991; Orth and Rothwell, 2004), measurement of cSP may be exceptionally suitable for longitudinal studies in patients, or before and after experimental manipulation. We conclude that the TMS evoked cortical excitability cSP measure can be safely tested in intrinsic laryngeal muscles in healthy volunteers. The use of fine-wire electrodes to measure the cSP of the LMC as validated in this work provides a tool to evaluate GABA receptor mediated inhibition process in the LMC. This will enable the direct comparison of inhibitory responses in healthy individuals and people with neurological voice disorders, such as spasmodic dysphonia and voice tremor which in turn will lead to a better understanding of the pathophysiology of these conditions

## Author contributors

MC, experimental system design, methodology, data collection, data processing, data analysis, data interpretation, drafting the work, final approval of the version to be published; RS, conception of the study, data collection, data interpretation, drafting the work, final approval of the version to be published; GG, conception of the study, methodology, data collection, final approval of the version to be published; SS, methodology assistance, data interpretation, final approval of the version to be published; CL, methodology, data interpretation, final approval of the version to be published; CP, data collection, drafting the work, final approval of the version to be published; TK, conception of the study, methodology, data collection, data analysis, data interpretation, drafting the work, final approval of the version to be published.

## Funding

This work was partly supported by the National Institute of Communication Disorders and Deafness, National Institutes of Health (grant number R21DC012344), the University of Minnesota's MnDRIVE (Minnesota's Discovery, Research and Innovation Economy) initiative, the National Center for Advancing Translational Sciences of the National Institutes of Health (award number UL1TR000114) and, National Institute of Biomedical Imaging and Bioengineering (grant number P41 EB015894).

### Conflict of interest statement

The authors declare that the research was conducted in the absence of any commercial or financial relationships that could be construed as a potential conflict of interest.
