# Supplementary material for: Evaluation of the Cortical Silent Period of the Laryngeal Motor Cortex in Healthy Individuals
Source: Front Neurosci. 2017 Mar 7;11:88. doi: 10.3389/fnins.2017.00088 (PMC5339278; doi:10.3389/fnins.2017.00088)
Supplement: Supplementary file 1 [file Table1.docx]

| Table 1. Laryngeal motor cortex hotspot coordinates | | | | | | |
| --- | --- | --- | --- | --- | --- | --- |
| Participant | Left LMC | | | Right LMC | | |
|  | x | y | z | x | y | z |
| 1 | -61 | -2 | 23 | 67 | -10 | 18 |
| 2 | -61 | -9 | 36 | 59 | -15 | 41 |
| 3 | -61 | -3 | 32 | 53 | 3 | 47 |
| 4 | -58 | 1 | 28 | 60 | 17 | 14 |
| 5 | -61 | -7 | 18 | 59 | 5 | 26 |
| 6 | -57 | 0 | 36 | 64 | 6 | 13 |
| 7 | -54 | -8 | 50 | 52 | -19 | 58 |
| 8 | -52 | 5 | 43 | 49 | 11 | 41 |
| 9 | -61 | 0 | 31 | 50 | 14 | 42 |
| 10 | -54 | 3 | 44 | 55 | 2 | 42 |
| 11 | -35 | -8 | 55 | 47 | 14 | 61 |
| Average | -56 | -3 | 36 | 56 | 3 | 37 |
| All coordinates are the perpendicular projection of the TMS coil center on the surface of the motor cortex. Coordinates values are reported in MNI standard space. Average location was calculated based on the center of mass algorithm. LMC: laryngeal motor cortex. | | | | | | |
