# Supplementary material for: Evaluation of the Cortical Silent Period of the Laryngeal Motor Cortex in Healthy Individuals
Source: Front Neurosci. 2017 Mar 7;11:88. doi: 10.3389/fnins.2017.00088 (PMC5339278; doi:10.3389/fnins.2017.00088)
Supplement: Supplementary file 2 [file Table2.docx]

| Table 2. Peripheral stimulation evoked potential latency | | | | |
| --- | --- | --- | --- | --- |
| Participant | Resting mean±SD | | Active mean±SD | |
|  | L-TA | R-TA | L-TA | R-TA |
| 1 | 10.3±0.0 | 4.4±0.1 | 10.3±0.1 | 4.8±0.0 |
| 2 | 7.8±0.1 | 5.6±0.1 | 7.6±0.2 | 5.7±0.2 |
| 3 | 7.8±0.1 | 6.1±0.1 | 7.1±0.1 | 6.0±0.1 |
| 4 | 10.1±0.1 | 7.8±0.1 | 9.9±1.0 | 7.3±0.0 |
| 5 | 7.4±0.1 | 6.9±0.5 | 8.6±0.3 | 7.0±0.3 |
| 6 | 5.7±0.1 | 5.7±0.2 | 5.9±0.4 | 5.7±0.9 |
| 7 | 7.6±0.2 | 5.1±0.1 | 8.8±0.5 | 4.7±0.3 |
| 8 | N/A | 8.9±0.1 | N/A | 8.5±0.1 |
| 9 | 12.8±0.1 | 8.9±0.1 | 12.9±0.1 | 8.3±0.2 |
| 10 | 12.8±0.1 | 8.1±1.0 | 12.8±0.4 | 8.5±0.4 |
| 11 | 9.0±0.1 | 6.1±0.5 | 8.6±0.4 | 6.4±0.5 |
| Latency values were calculated based on the first peak of the evoked potential. L-TA: left thyroarytenoid. R-TA: right thyroarytenoid. | | | | |
